# Prevalence and predictors of pornography exposure during the third wave of the COVID-19 pandemic: A web-based cross-sectional study on students in Bangladesh

**DOI:** 10.3389/fpubh.2022.1046813

**Published:** 2022-12-20

**Authors:** Md. Tanvir Hossain, Benojir Ahammed, Nusrat Jahan, Md. Akhtarul Islam, Md. Mostafizur Rahman, Bayezid Khan, Md. Juwel Ahmed Sarker, Md. Mahdi-Al-Muhtasim Nibir, Mahamudul Hasan, Mir Hasib, Rumana Rahman, Md. Nazrul Islam

**Affiliations:** ^1^Sociology Discipline, Social Science School, Khulna University, Khulna, Bangladesh; ^2^Statistics Discipline, Science, Engineering and Technology School, Khulna University, Khulna, Bangladesh; ^3^Department of Disaster Management and Resilience, Faculty of Arts and Social Sciences, Bangladesh University of Professionals, Dhaka, Bangladesh; ^4^Development Studies Discipline, Social Science School, Khulna University, Khulna, Bangladesh; ^5^Department of Development Studies, Faculty of Social Science and Humanities, Hajee Mohammad Danesh Science and Technology University, Dinajpur, Bangladesh; ^6^Mass Communication and Journalism Discipline, Social Science School, Khulna University, Khulna, Bangladesh; ^7^English Discipline, Arts and Humanities School, Khulna University, Khulna, Bangladesh; ^8^Forestry and Wood Technology Discipline, Life Science School, Khulna University, Khulna, Bangladesh

**Keywords:** prevalence, pornography exposure, risk factors, COVID-19, students, Bangladesh

## Abstract

**Background:**

Pornography exposure, particularly among students, in Bangladesh, has increased in the twenty-first century. However, pornography exposure during the COVID-19 pandemic, when people were compelled to “stay at home” and relied extensively on the internet for all forms of activities, including academia, socializing, and communication, has remained unexplored. The present study aimed to assess the prevalence of pornography exposure among students during the third wave of the COVID-19 pandemic and to determine the associated predictors.

**Methods:**

A web-based cross-sectional study was carried out among students with certain specifications, i.e., current students at high school/college/university with access to the internet and valid social media accounts. By administering a semi-structured e-questionnaire using Google Forms, a total of 646 valid responses were retained for this study. The data were analyzed in two phases by Pearson's Chi-square and multiple logistic regression model, using IBM SPSS Statistics, version 25. The results were expressed as an adjusted odds ratio (AOR) with a 95% confidence interval (95% CI).

**Results:**

The findings suggest that 75.9% of students were exposed to pornography during the third wave of the COVID-19 pandemic and preferred to watch the amateur/professional genre of pornography. Pornography exposure was significantly associated with age and relationship status, as students aged 22–24 years (95% CI: 1.01–2.41; *p* = 0.045) and over 25 years (95% CI: 1.61–10.03; *p* = 0.003) were more likely to watch pornography, while married students and those in relationships (95% CI: 1.24–3.49; *p* = 0.006) also watched pornography during the pandemic. In contrast, students who were living alone (95% CI: 0.38–0.92; *p* = 0.021), were living in the Khulna division (95% CI: 0.16–0.52; *p* < 0.001) or had a negative attitude toward pornography (95% CI: 0.94–0.99; *p* = 0.002) were less likely to be exposed to pornography.

**Conclusion:**

Pornography exposure was higher among students in Bangladesh during the COVID-19 pandemic, and religiosity and disapproving attitudes toward pornography negatively influenced the pornography exposure. For a better understanding of the complex dynamics of socio-demographic issues with pornography exposure among students, extensive research is required for policymakers to devise appropriate strategies and interventions to ensure healthy and safe sex life for the younger population.

## Introduction

The outbreak of coronavirus disease (COVID-19) in late 2019 in Wuhan, China (1) and the declaration of a pandemic in mid-March 2020 by the World Health Organization (2) necessitated drastic non-therapeutic protective and preventive measures, i.e., “lockdown,” “social distancing,” and “face mask,” for the general population and “home confinement,” “isolation,” and “quarantine” for infected and suspected people. This was done in order to contain the virus and to minimize the risk of “human-to-human” infection (3, 4). Despite the collective efforts of governments and international organizations, the world has witnessed over 60 million confirmed cases and 6.5 million deaths as of 7 September 2022 (5).

The prolonged “lockdown” adversely affected people's mental wellbeing, and a spike in mental health disorders was observed worldwide (6, 7); people in Bangladesh were no exception. Studies have suggested that people, irrespective of age, sex, caste, and creed, have experienced an unprecedented increase in mental health disorders and associated self-harm behaviors, including depression, anxiety, stress, and fear (8–13). This may have led to sleep disturbance (14) and in the worst cases suicide (15, 16), particularly among students. In addition to degraded mental health during the “lockdown,” students were also frustrated due to uncertainty over their academic future and professional career (17), although the government did begin online/remote education in order to continue educational activities (18) while maintaining “social distancing.” However, during the “lockdown,” online activities increased substantially among students. For example, Hossain et al. (19) reported that during the first wave of COVID-19 in Bangladesh, 64.1% of 493 students were almost always active in their “virtual life.” A similar study in China also noticed that 87.1% of the 1,189 students frequently updated their online statuses (20).

To maintain social and relational communication and to be involved in different events, including academia, socializing, leisure, and shopping, young people did almost everything in their “virtual life” (21); they, thus, became obsessed with a wide range of online-based activities during “lockdown”, one of which was watching pornography (21–25). Zamboni et al. (21), for example, reported that 21.6% of Italian adults were using pornographic materials during the “lockdown.” A similar study on adults in the United Kingdom and the United States suggests that, on average, pornography viewing increased during the lockdown, from 5.54 to 8.55 h per week (23). In a study on health service providers in New Zealand, Rodda et al. (24) noted that pornography viewing among health service recipients increased significantly during the period of social distancing.

Regarding pornography exposure, there have been a few studies undertaken in Bangladesh. Mamun et al. (26), for example, found that 72% of 313 undergraduate students had consumed pornography at least once in their lifetime, and half of them consumed it frequently. They further noted that pornography exposure was positively determined largely by male sex, urban residence, involvement in romantic relationship, and frequent use of social media, while negative attitudes toward pornography did not impact on pornography exposure (26). Golder et al. (27), in their study, found that 81% of students had been exposed to pornography before 16 years of age and that pornography exposure was significantly associated with residence, religiosity, and internet access. A similar study on private university students observed that 41.8% of students watched porn, and that the prevalence was significantly higher among male students (28). From the existing literature, it is evident that there is a lack of empirical research on pornography exposure among students during the COVID-19 pandemic in Bangladesh. The current study aims to assess the prevalence and predictors of pornography exposure during the pandemic and to fill the literature gap by exploring the phenomenon nationwide.

## Materials and methods

### Study settings and participants

**Figure 1:**
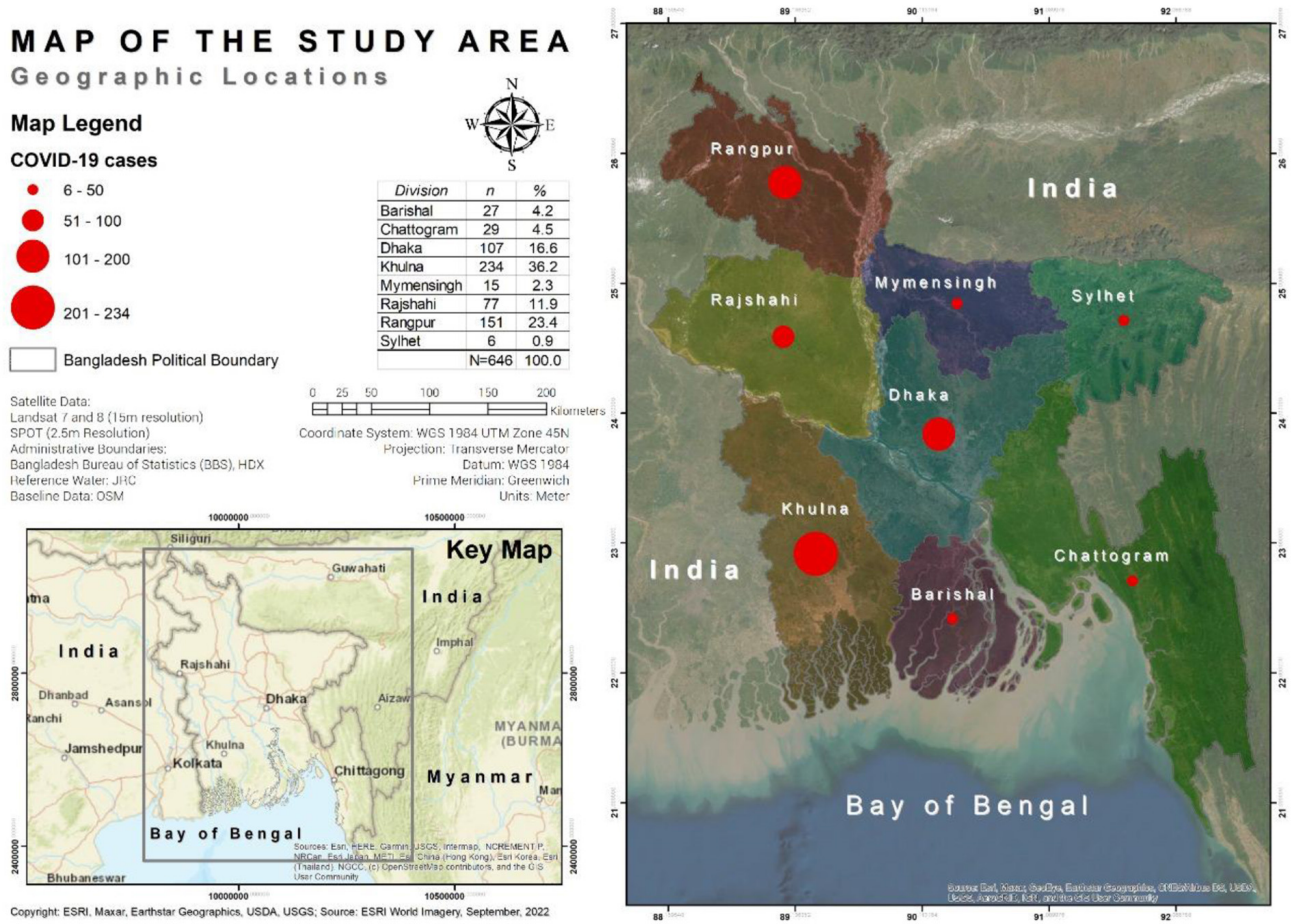
Map of the study area.

This web-based cross-sectional explanatory study was administered through a semi-structured e-questionnaire using Google Forms among students during the COVID-19 pandemic in all eight divisions of Bangladesh (see Figure 1). The participants were approached based on certain inclusion criteria: (i) must be students; (ii) must be enrolled in either school (school students must be in either Class IX or Class X), college or university; (iii) must have access to the internet through desktop, laptop, tablet, or smartphone; (iv) must have an active account on social media (Facebook, Messenger, or WhatsApp). Accounting for the above criteria, around 1,200 potential participants were purposively approached through social media by their respective school, college or university teachers, and the link to the e-questionnaire was shared alongside the online written informed consent form. The semi-structured e-questionnaire was divided into five mutually exclusive modules, focusing mainly on socio-demographic information, pornography exposure during the COVID-19 pandemic, and attitudes toward pornography, respectively. The e-questionnaire was in English and Bangla in order to allow the participants to comprehend the questions. The data were collected from June to July 2022, and out of a total of 662 anonymous responses, the information from 646 participants was retained for this study after careful scrutiny; the rest (16 responses) were discarded because of repetitive responses and missing information.

### Ethical issues

This study was approved by the institutional ethical clearance committee (Reference No. KUECC – 2022/06/15). The participants responded to this web-based cross-sectional study by filling out a written informed consent form in the first section of the e-questionnaire. All participants responding voluntarily to the e-questionnaire were provided with information in the consent form which explained the research purpose, anonymity, confidentiality of information, and right to revoke participation without prior justification.

### Measures

#### Socio-demographic information

The socio-demographic information consisted of age (in year), sex (male, others—female and third sex), education (honors, others—higher secondary and master), religious preference (atheist/agnostic, Muslim, others—Hindu, Christian, and Buddhist), self-rated religiousness (below average, average, above average) (29) residence (rural/suburban, urban), relationship status (unmarried/single, married/in a relationship), socioeconomic status (marginal/lower class, middle/upper class) (9, 30), living arrangement (with roommates/romantic partner, with parents/spouse, alone), division (Dhaka, Khulna, Rangpur, others—Barishal, Chattogram, Mymensingh, and Sylhet), sexual orientation (homosexual/bisexual, heterosexual) and educational institutions (public university, others—college, national university, and private university).

#### Pornography exposure

Pornography exposure in Bangladesh was assessed as per previous studies (26–28). The questions regarding pornography exposure during COVID-19 were—“Did you watch pornography in the past two months during COVID-19?”, and “What type of pornography did you most prefer to watch?” In this study, pornography is defined as “any sexually explicit content, whether videos or pictures on the internet, which showed intimate relations ‘between men and women' or between women' or ‘between men' and led to sexual arousal among the viewers”.

#### Attitude toward pornography

The attitude toward pornography (AtP) is a scale developed by Evans-DeCicco et al. (31) to measure general positive and negative attitudes toward pornography. The AtP consists of 13 items and is scored on a 7-point Likert scale, ranging from “strongly disagree” to “strongly agree,” with a higher score indicating an anti-pornography attitude (31). The overall internal consistency of the original AtP was 0.85. In this study, the AtP was measured on a five-point Likert scale, and the internal consistency was Cronbach's α = 0.78.

### Analysis

The data were analyzed in two consecutive stages using IBM SPSS Statistics, version 25. First, the Pearson's Chi-square (χ^2^) test of independence was executed to measure the association of the explanatory variables with pornography exposure at 5% level of significance; at the second and final stage, the multiple logistic regression model was performed accounting for the pornography exposure-related variables found to be statistically significant in the Pearson's Chi-square (χ2) test. The findings are shown using the adjusted odds ratio (AOR) with a 95% confidence interval (95% CI).

## Results

Table 1 presents the prevalence of pornography exposure and the basic characteristics of the participants. The overall prevalence of pornography exposure was 75.9%. Regarding the socio-demographic characteristics among the participants, the majority (55.6%) were aged 22–24 years, and 72.0% were male students. Most of the participants (84.7%) were undergraduate students and 80.3% were Muslim. More than half (57.4%) of the participants lived in urban areas, and 74.0% were unmarried/single; 80.7% came from middle/upper class families and 92% studied in public universities.

**Table 1:** Basic characteristics of participants and their association with pornography exposure.

**Variables**	***f* (%)**	**Pornography exposure**		**Test statistics**	***p*-value**
		**No**	**Yes**		
**Overall**		**156 (24.1)**	**490 (75.9)**		
Age					
≤ 21	173 (26.8)	65 (37.6)	108 (62.4)		
22–24	359 (55.6)	83 (23.1)	276 (76.9)	35.491	**< 0.001**
25≥	114 (17.6)	8 (7.0)	106 (93.0)		
Sex					
Others^a^	181 (28.0)	56 (30.9)	125 (69.1)	6.330	**0.012**
Male	465 (72.0)	100 (21.5)	365 (78.5)		
Education					
Others^b^	99 (15.3)	11 (11.1)	88 (88.9)	10.850	**0.001**
Honors	547 (84.7)	145 (26.5)	402 (73.5)		
Religious preference					
Atheist/agnostic	31 (4.8)	1 (3.2)	30 (96.8)		
Others^c^	96 (14.9)	21 (21.9)	75 (78.1)	8.470	**0.014**
Muslim	519 (80.3)	134 (25.8)	385 (74.2)		
Self-rated religiousness					
Below average	82 (12.7)	8 (9.8)	74 (90.2)		
Average	219 (33.9)	51 (23.3)	168 (76.7)	12.326	**0.002**
Above average	345 (53.4)	97 (28.1)	248 (71.9)		
Residence					
Rural/sub-urban	275 (42.6)	58 (21.1)	217 (78.9)	2.444	0.118
Urban	371 (57.4)	98 (26.4)	273 (73.6)		
Relationship status					
Unmarried/single	478 (74.0)	132 (27.6)	346 (72.4)	12.058	**0.001**
Married/in a relationship	168 (26.0)	24 (14.3)	144 (85.7)		
Socioeconomic status					
Marginal/lower class	125 (19.3)	19 (15.2)	106 (84.8)	6.776	**0.009**
Middle/upper class	521 (80.7)	137 (26.3)	384 (73.7)		
Living arrangement					
With roommates/romantic partner	344 (53.3)	69 (21.1)	275 (79.9)		
With parents/spouse	212 (32.8)	69 (32.5)	143 (67.5)	12.152	**0.002**
Alone	90 (13.9)	18 (20.0)	72 (80.0)		
Division					
Others^d^	154 (23.8)	20 (13.0)	134 (87.0)		
Dhaka	107 (16.6)	23 (21.5)	84 (78.5)	32.416	**<0.001**
Khulna	234 (36.2)	85 (36.3)	149 (63.7)		
Rangpur	151 (23.4)	28 (18.5)	123 (81.5)		
Sexual orientation					
Homosexual/bisexual	28 (4.3)	1 (3.6)	27 (96.4)	6.766	**0.009**
Heterosexual	618 (95.7)	155 (25.1)	463 (74.9)		
Educational institution					
Others^e^	52 (8.0)	8 (15.4)	44 (84.6)	2.371	0.124
Public university	594 (92.0)	148 (24.9)	446 (75.1)		

*f*, Frequency.

a Others include female and third sex.

b Others include secondary, higher secondary and master.

c Others include Hindu, Christian, and Buddhist.

d Others include Barishal, Chattogram, Mymensingh, and Sylhet.

e Others include college, national university, and private university.

Bold values are significant at 5% level of significance.

According to Table 2, pornography exposure increased with the increase in age [ranging from 62.4% (<21 years) to 93.0% (25 ≥ years)]; male participants (78.5%) were more exposed to pornography than other sexes. It was also found that honors students (73.5%) were less exposed to pornography than other students (88.9%), while atheists and agnostics had a higher (96.8%) exposure to pornography than Muslims (74.2%) and other religious groups (78.1%). Regarding residence, pornography exposure was relatively higher among rural and sub-urban students (78.9%) than among those from urban areas (73.6%). About 86% of students who were married or in a relationship watched pornography; pornography exposure was also relatively high among students from marginal/lower class families (84.8%). Students living alone (80.0%) or with roommates and romantic partners (79.9%) had more exposure to pornography than those living with parents or families (67.5%). The percentage of pornography exposure was higher among college, national university, and private university students (84.6%).

**Table 2:** Multiple logistic regression analysis of variables associated with exposure to pornography.

**Variables**	**B**	**SE**	**Sig**.	**AOR Exp (B)**	**95% CI of AOR**	
					**Lower**	**Upper**
Age						
≤ 21^RC^				1.00		
22–24	0.445	0.222	**0.045**	1.56	1.01	2.41
25≥	1.392	0.466	**0.003**	4.02	1.61	10.03
Sex						
Others^a^^RC^				1.00		
Male	0.309	0.222	0.164	1.36	0.88	2.11
Education						
Others^b^^RC^				1.00		
Honors	−0.329	0.401	0.413	0.72	0.33	1.58
Religious preference						
Atheist/agnostic^RC^				1.00		
Others^c^	−1.381	1.115	0.216	0.25	0.03	2.24
Muslim	−1.509	1.090	0.166	0.22	0.03	1.87
Self-rated religiousness						
Below average^RC^				1.00		
Average	−0.762	0.448	0.089	0.47	0.19	1.12
Above average	−0.955	0.436	**0.029**	0.39	0.16	0.91
Relationship status						
Unmarried/single^RC^				1.00		
Married/in a relationship	0.732	0.264	**0.006**	2.08	1.24	3.49
Socioeconomic status						
Marginal/lower class^RC^				1.00		
Middle/upper class	−0.422	0.295	0.152	0.66	0.37	1.17
Living arrangement						
With roommates/romantic partner				1.00		
With parents/spouse	−0.186	0.323	0.564	0.83	0.44	1.56
Alone	−0.518	0.224	**0.021**	0.60	0.38	0.92
Division						
Others^d^^RC^				1.00		
Dhaka	−0.515	0.362	0.155	0.60	0.29	1.22
Khulna	−1.239	0.297	**< 0.001**	0.29	0.16	0.52
Rangpur	−0.489	0.336	0.146	0.61	0.32	1.19
Sexual orientation						
Homosexual/bisexual^RC^				1.00		
Heterosexual	−1.508	1.050	0.151	0.22	0.03	1.73
Attitude toward pornography	−0.038	0.012	**0.002**	0.96	0.94	0.99

B, unstandardized regression weight; SE, standard error; Sig., significance; AOR, adjusted odds ratio; Exp (B), predicted change in odds for an increase in the predictor; CI, confidence interval. Bold values are significant at 5% level of significance.

a Others include female and third sex.

b Others include secondary and master.

c Others include Hindu, Christian, and Buddhist.

d Others include Barishal, Chattogram, Mymensingh, and Sylhet.

e Others include college, national university and private university.

RC Reference category.

In bivariate analysis, factors such as age (*p* < 0.001), sex (*p* = 0.012), education (*p* = 0.001), religious preference (*p* = 0.014), self-rated religiousness (*p* = 0.002), relationship status (*p* = 0.001), socioeconomic status (*p* = 0.009), living arrangement (*p* = 0.002), division (*p* < 0.001), and sexual orientation (*p* = 0.009) were significantly associated with pornography exposure.

**Figure 2:**
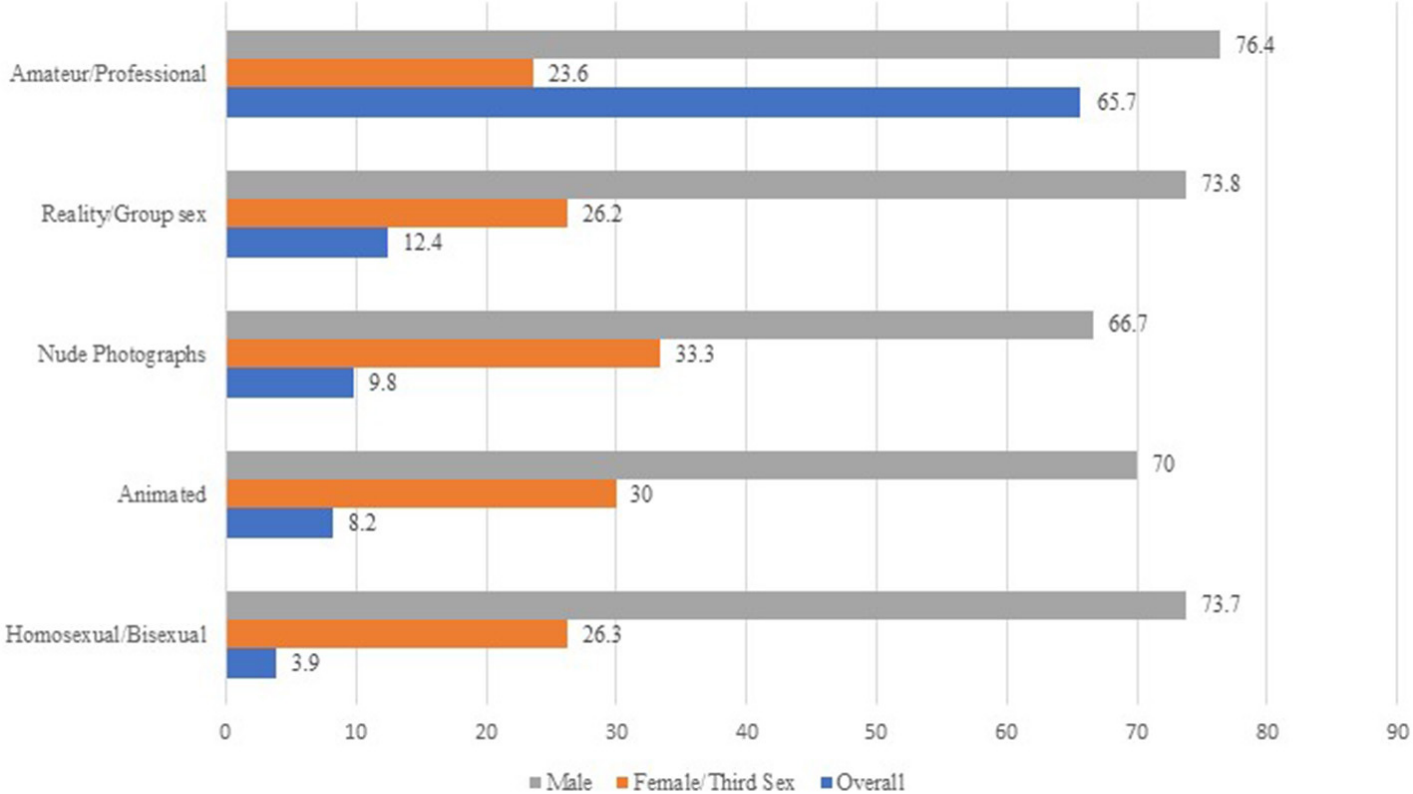
Type of pornography preferred by sexes (*N* = 490).

Figure 2 suggests the types of pornography preferred by the sexes. Findings showed that three out of five students watched amateur or professional pornography (65.7%), of these, three quarters were male (76.4%) and the rest were female (23.6%). However, only 3.9% of participants viewed homosexual/bisexual pornography; female participants constituted a quarter of these viewers (26.3%).

From the bivariate analysis (Table 1), this study only considered significant factors for an adjusted binary logistic regression model, measured by the odds ratio with a 95% confidence interval. The findings suggested that students aged 22–24 years and over 25 years were 1.56 (95% CI: 1.01–2.41; *p* = 0.045) and 4.02 (95% CI: 1.61–10.03; *p* = 0.003) times more likely, respectively, to report watching pornography in the past 2 months compared to students aged 21 and under. Students with above-average religiousness were 0.39 (95% CI: 0.16–0.91; *p* = 0.029) times less likely to report watching pornography than those with below-average religiousness. It is also apparent that married students and those in relationships were 2.08 (95% CI: 1.24–3.49; *p* = 0.006) times more likely to report watching pornography in the past 2 months compared to students who were unmarried or single. In contrast, students who were living alone, students living in Khulna division and students with an anti-pornography attitude were 0.60 (95% CI: 0.38–0.92; *p* = 0.021), 0.29 (95% CI: 0.16–0.52; *p* < 0.001), and 0.96 (95% CI: 0.94–0.99; *p* = 0.002) times less likely, respectively, to report watching pornography.

## Discussion

The study aimed to explore the prevalence of pornography exposure among students and to identify its predictors during the third wave of the COVID-19 pandemic in Bangladesh. The results showed that three out of four students admitted to watching pornography in the last 2 months during the COVID-19 pandemic. The overall pornography exposure reported in this study during the COVID-19 pandemic was higher than that of Italy (21.6%) (21), the USA (21%) (32), the UK (43%) (23), and New Zealand (30%) (24), and significantly higher than reported by pre-COVID-19 studies in Bangladesh [42% (28), and 72% (26)]. The heightened prevalence of pornography among students can be attributed to the growing number of internet users during COVID-19 (33), particularly among the younger population, who had more access to and better familiarity with the internet (22, 26).

This study observed that students above 21 years of age watched pornography higher than their younger counterparts during the COVID-19 pandemic. A possible reason for higher pornography exposure could be their inclination to learn about and prepare for sexual activities during marriage, as the average age at marriage for men in Bangladesh is 25.2 years, for women it is 19.4 years (34). Besides, watching pornography for sex education was not an exception (35). In contrast, students under 21 may have been spending more time with their peers in offline activities, minimizing online engagement and reducing exposure to pornography (36). The findings of the current study complement the work of Golder et al. (27) who noted a higher exposure to pornography among older students, while Chowdhury et al. (28) found greater exposure among younger students than among their older counterparts.

The current study found that students with above-average religiosity were less exposed to pornography during the pandemic. A sense of moral stigma around pornography (37), together with a fear of shame on the part of religious persons, may have deterred them from watching pornography (38). Eljawad et al. (39), in explaining the lower prevalence of pornography searches on the internet among Arabs, pointed to the high degree of religiosity and conservatism in the Muslim-dominated Middle Eastern countries, which do not approve of sexual activities outside marriage. Likewise, Bangladesh—a Muslim majority country—does not promote sexual content online or offline; additionally, it endorsed an act to reduce people's exposure to pornography across the country (40), because exposure to pornography may lead to offensive sexual behavior toward women. Vera Cruz (41), for example, found that men with frequent exposure to pornography, especially young men, were more likely to engage in sadistic sexual behavior toward their female partners, largely due to their sexual fantasies. However, the findings of this study supports that of Golder et al. (27), who noted that higher religiosity reduces the possibility of pornography exposure; Mamun et al. (26), in contrast, found no influence of religious and moral values on pornography consumption, because strong religiosity may not always translate into specific activities prohibited by religion, such as pornography use (42).

Married students or those in a relationship watched pornography higher than those of unmarried and single students during the pandemic; this finding complements the work of Cascalheira et al. (43) as they observed an increase in sexual fantasies among people living with romantic partners during the “lockdown.” The finding is also aligned with pre-COVID studies in Bangladesh (26) and elsewhere (38). Intensified exposure to pornography among people in relationships can be attributed to the fact that watching pornography with a spouse or romantic partner may increase willingness to explore newer sexual practices in order to meet each other's sexual wants and fantasies. Some studies, however, suggest otherwise. Perry (44), for example, in a longitudinal study, found that frequent exposure to pornography may not only reduce sexual satisfaction but also degrade the quality of marital life, largely because of unmet sexual fantasies and increasingly sadistic sexual behavior (41).

In addition, it is apparent that students living alone watched pornography less than those living with roommates or romantic partners. This lower exposure to pornography can be attributed to these students' more frequent involvement in peer groups engaged in offline activities (36), as well as the growing pressure of the online education system during COVID-19 (17, 18), which may have occupied their free time. Cascalheira et al. (43) and Sallie et al. (23), in contrast, found an increase in sexual practice and pornography consumption among people living alone; this behavior was attributed to boredom and increased free time. Rothman et al. (35) also observed that loneliness or boredom were the key factors motivating young Americans, both men and women, to watch pornography and thus satisfy their sexual desires through masturbation.

Regarding residence, it appeared that students from the Khulna division had lower exposure to pornography; their limited access to computers and the internet may have reduced their exposure. The latest nationwide survey on ICT use and access by individuals and households has shown that only 2.3% of households in the Khulna division have a computer, with another 4.2% having access to the internet (45). Although there was a sharp rise in internet users during COVID-19 (33), the unstable internet connectivity, frequent load shedding, and inadequate low-cost devices (18, 46) may have limited access and exposure to pornography among students in the Khulna division. Furthermore, financial struggle cannot be denied, particularly among middle and lower-income people in the southwestern region of Bangladesh (9, 47–49); this may also have had some impact on access to the internet and exposure to pornography during the COVID-19 pandemic.

Attitudes toward pornography also predict the pornography exposure, as students with anti-pornography attitudes were less exposed to pornography; this result supports a pre-COVID-19 study in Bangladesh. Mamun et al. (26) observed that a positive attitude toward pornography may have increased unhealthy practices and sentiments among university students, while a negative attitude could prevent them from engaging in sadistic behavior, particularly against the opposite sex. Likewise, Evans-DeCicco and Cowan (31) found that anti-pornography attitudes are significantly associated with negative views about pornography and its actors.

## Strengths and limitations

The current study has certain strong points. First, to the best of the authors' knowledge, this is the only empirical study that has investigated the prevalence and predictors of pornography exposure among students during the COVID-19 pandemic in Bangladesh. Second, this study was carried out using an online platform, avoiding the risk of “human-to-human” infection with COVID-19 by maintaining “social distancing.” Third, the data regarding pornography exposure among students were collected through a globally approved and reliable standardized questionnaire. Nevertheless, no study is without limitations. This was a cross-sectional study; therefore, causality could not be established. Data were only collected from students with access to the internet, which may limit the generalizability of the findings to other groups. The sample selection, using non-probability sampling, may also have limited the generalizability due to sampling biases. The self-evaluation of pornography exposure by students may have played a role in over- or under-reporting pornography exposure. Moreover, the adjusted models identified some crucial predictors of students' pornography exposure; nevertheless, there may be a possibility of residual confounding. As such, more extensive research on a nationally representative population is required.

## Conclusions and policy implications

The current study aimed to assess the prevalence and identify the predictors of pornography exposure among students during the COVID-19 pandemic. The findings showed that pornography exposure was higher among students than in previous studies conducted before COVID-19; various factors, including age, religiosity, relationship status, living arrangement, residence, and attitude, significantly predicted pornography exposure. For a better understanding of the complex dynamics of socio-demographic issues with pornography exposure among students, extensive research (longitudinal as well as in-depth studies) is required for policymakers to devise appropriate strategies, e.g., introducing sex education at secondary and higher secondary levels, to ensure healthy and safe sex life for the younger population through age-specific interventions.

## Data availability statement

The raw data supporting the conclusions of this article will be made available by the authors, without undue reservation.

## Ethics statement

The studies involving human participants were reviewed and approved by Khulna University Ethical Clearance Committee Khulna University. Written informed consent to participate in this study was provided by the participants.

## Author contributions

MTH: conceptualization, investigation, data curation, formal analysis, methodology, resources, software, and writing—original draft. BA, NJ, MAI, and MR: data curation, formal analysis, software, and writing—original draft. BA, MAI, MR, BK, MS, MN, MHasa, MHasi, and MNI: investigation and resources. BK, MS, MN, and RR: resources and writing—original draft. All authors: writing—review and editing. All authors contributed to the article and approved the submitted version.
